# Modulatory potential of A novel benzofuran-derived compound against aluminium chloride-induced neurotoxicity and miRNA dysregulation: Biochemical and bio-analytical study

**DOI:** 10.1016/j.toxrep.2025.102148

**Published:** 2025-10-25

**Authors:** Maha Zaki Rizk, Ghadha Ibrahim Fouad, Eman Younis, Khalda Amr, Nesma Elaraby, Maha Fawzi Emam, Aya Rashad Abdou, Nesrin Fouad Taha, Laila Hasanin Emara, Hanan Farouk Aly

**Affiliations:** a Department of Therapeutic Chemistry, Pharmaceutical and Drug Industries Research Institute, National Research Centre, 33 El-Bohouth St., Dokki, Cairo P.O. 12622, Egypt; b Medical Molecular Genetics Department, National Research Centre, 33 El-Bohouth St., Dokki, Cairo P.O. 12622, Egypt; c Medicinal and Pharmaceutical Chemistry Department, Pharmaceutical and Drug Industries Research Institute, National Research Centre, 33 El-Bohouth St., Dokki, Cairo P.O. 12622, Egypt

**Keywords:** AlCl_3_-induced neurotoxicity, Neuroinflammation, UHPLC/UV, Bio-analytical method development, miRNA-15a, miRNA-132, miRNA-34a

## Abstract

The aim of the current work was to investigate the neuro-modulatory activities of a novel synthesized compound, namely, 3-((3-Acetylphenyl) amino)-1-(benzofuran-2-yl) prop-2-en-1-one (designated as **compound IV**) in aluminium-chloride (AlCl3)-intoxicated rats. Moreover, for the first time, quantitative analysis of compound-IV in rat plasma was developed using a novel, properly validated Ultra-High-Performance-Liquid Chromatography / Ultra-Violet (UHPLC/UV) method. For the biochemical studies; four-groups were included: negative-control, AlCl_3_-intoxicated-rats, intoxicated-rats treated with compound-IV, and reference-donepezil, respectively. Biochemical/molecular assays were conducted; levels of interleukin-6 (IL-6), total antioxidant-capacity (TAC), brain-derived-neurotrophic-factor (BDNF), and total protein content (TP). Differential expressions of miRNA-34a, miRNA-15a, and miRNA-132, were assessed. For the bio-analytical studies; several chromatographic-conditions and extraction-procedures were meticulously optimized. Biochemical results revealed that AlCl_3_ (a neurotoxic-agent), enhanced neuroinflammation, oxidative-stress and synaptic-dysfunction; as indicated by increased IL-6 levels, declined both TAC levels and BDNF contents. Moreover, significant dysregulation in miR-34a, −15a, and −132 levels were observed. In contrast, treatment of neuro-intoxicated rats with compound-IV ameliorated all the investigated biomarkers. This novel-benzofuran-derivative exerts its neurotherapeutic-activity by reducing AlCl_3_-induced neurotoxicity and mitigating oxidative-stress, neuroinflammation, and synaptic-dysfunction through regulating all miRNA levels. The developed and validated analytical-method ensured that best quantitative separation of compound-IV was achieved using Symmetry-C18-column, with mobile phase consisting of acetonitrile: H_2_O (50: 50), UV detection at λmax 390-nm, 1 mL/min flow-rate and 3.4 min retention time. The proposed method provides excellent specificity and linearity over concentration range of 1–100 μg/mL; hence, it could serve as a perquisite-step for further investigation of bioavailability (BA) and pharmacokinetics (PKs) of this compound.

## Highlights

• Aluminium-chloride (AlCl_3_) enhances neuroinflammation and synaptic dysfunction.
• AlCl_3_ stimulated miRNA dysregulation including miRNA-34a, miRNA-15a, and miRNA-132.
• **Compound (IV)** demonstrated therapeutic potential against AlCl_3_-induced neurotoxicity.
• The UHPLC/uv method offers a precise, low-cost technique to quantify **Compound (IV)**
• The method may enable future estimation of primary and secondary PK parameters

## Graphical Abstract

**Figure:**
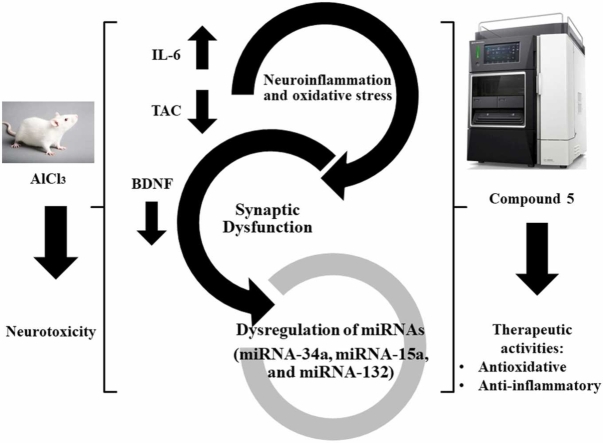

## 1 Introduction

Amongst the environmental factors, the aluminum (Al) toxicity has been associated with the increasing incidence of Alzheimer’s disease (AD)-like neuropathology. Al is one of the most widely distributed elements of the earth's crust (about 8 %). Humans are exposed massively to Al from contaminated food and beverages, air pollution, and water pollution, in addition, Al could be found in aluminum utensils, aluminum foils, water treatment, food additives, pharmaceutical preparations (*e.g.,* antacids phosphate binders, vaccines, and buffer formulations) and cosmetics [1], [2]. The neurotoxic manifestations of Al could be ascribed to its high potential to stimulate oxidative stress along with mitochondrial dysfunction, apoptosis, synaptic dysfunction, and neuroinflammation in different brain regions mainly the cerebral cortex and the hippocampus [1], [3], [4]. Experimentally, Al-exposed animals demonstrated the development of both neurofibrillary tangles and amyloid (Aβ) plaques in the brain, which are the hallmarks of AD [1]. This was based on the association between Al and deposition of Aβ plaques in neurodegenerative disorders [5], [6], [7]. Furthermore, cholinergic system is highly vulnerable to Al toxicity, as previously reported in our studies [3], [8]; where Al activates acetylcholinesterase (AChE) activity in rodent and Drosophila models [3], [8], [9], [10]. This indicates the fact that the brain could be the main target for Al-triggered neurotoxicity [4]. Accordingly, further research is required to reveal the exact neurotoxic mechanism of Al in mediating neurodegeneration.

Several neurodegenerative disorders may result from toxic exposures or from certain neural diseases including Alzheimer’s disease (AD) and Parkinson’s disease (PD). MicroRNAs (miRNAs) are small non-coding RNA molecules that regulate genetic expression by binding to target messenger RNA (mRNA) molecules and promoting their degradation or suppressing their translation [11]. Dysregulation of several miRNAs is evident in the brains of affected patients or in neurodegeneration induced in experimental animals [11], [12]. Upregulation/downregulation of miRNA expression leads to alterations in the protein expressed by the corresponding pathogenic gene, which results in the development of neurodegenerative diseases [13]. Therefore, there is a need to explore the therapeutic potential and/or the diagnostic application of certain miRNAs in the process of the neuropathogenesis; thereby, up- or down-regulation of specific miRNAs would be a promising therapeutic approach that targets those miRNAs, for the treatment of neurodegenerative diseases [13].

Under the status of neurodegeneration, there is a complex relationship between oxidative stress and the expression of specific miRNAs; that leads to mitochondrial dysfunction and neuroinflammation [14], [15]. For instance, miR-15 can regulate cellular immunity and apoptotic pathways; therefore, it has a key role in the development of the neurodegenerative disorders [16]. In addition, mir-34 is a group that involves three domains: mir-34a, mir-34b, and mir-34c. The dysregulation of mir-34 is common in neurodegenerative disorders; mir-34c may play a role in neuronal signaling [17]. While, miR-34a is abundant in mature and differentiated neurotransmitters in mice, its expression and stimulation of nuclear factor 2-related factor 2 (NRF2) “the antioxidant protein expression regulator” demonstrated a neuroprotective impact against induced neurotoxicity *in vitro* [18]. Similarly, Hernandez-Rapp et al. [17] investigated the role of three miRNAs “mir-132, mir-124, and mir-34” implicated in cognitive impairment; mir-132 exhibited a regulatory role within the central nervous system (CNS) through controlling genes involved in neuronal plasticity and survival, and regulating synaptic proteins. MiR-132, identified as “NeurimmiR”, demonstrated a “negative regulatory effect” on inflammatory mediators [19]. Moreover, miR-132 has also been proposed as a member of a blood-based diagnostic biomarker for memory cognitive impairment (MCI) [20].

The expression of Brain-derived neurotrophic factor (BDNF) can be influenced by epigenetic alterations and upstream cytokines [21]. The parallel involvement of cytokines and BDNF in the etiology of neuroinflammatory conditions proposes that the impact of inflammation on brain function could be attributed to downstream impact on BDNF levels [22]. BDNF is mostly secreted by neurons and is found abundantly in different areas of the brain, but is able to cross the lood brain barrier (BBB) contributing to detectable blood levels [23]. *In vivo* studies showed that BDNF promotes central cholinergic neurotransmission, modulates neuronal plasticity, and protects against several neurodegenerative disorders by upregulating endogenous neuroprotective systems, such as antioxidant signaling pathways [8], [24]. Experimentally, a status of neurodegeneration might be stimulated by certain neurotoxic agents such as aluminium-chloride (AlCl_3_) [3], [24]; that is characterized by the depletion of acetylcholine (ACh) levels, AChE activation, deposition of neurotoxic Aβ plaques and tau proteins, and induction of oxidative stress and neuroinflammation [25]. In addition, exposure to Al compounds in animal models stimulates inflammatory responses, such as disturbance in certain cytokines especially interleukin-6 (Il-6), synaptic dysfunction and neurodegeneration in several brain regions [24].

Recently, developing reliable, suitable and non-invasive biomarkers for early diagnosis of neuroinflammation is essential to halt its progression [3]. Blood and cerebrospinal fluid (CSF) contain circulating miRNAs that could serve as accurate and predictive biomarkers in the diagnosis of AD pathogenesis. The expression levels of “miR-let-7b, miR-let-7e, miR-222, miR-206, miR-15a-5p, miR-let7i-5p, miR-613, miR-29a, and miR-125b were upregulated in AD patients, as compared to healthy controls [26]. Thereby, miRNAs have emerged as potential therapeutic targets for neurodegenerative disorders. In addition, these miRNAs could be used as circulating “molecular markers” through developing personalized “miRNA profiles” to enable efficient diagnosis of neurodegenerative diseases such as AD [27]. Unlike the more complex molecular neuroimaging techniques, analyzing miRNAs in bodily fluids offers a relatively simple and less invasive method for predicting AD [3], these remarkable miRNAs could be used as effective diagnostic markers and therapeutics for many progressive neurodegenerative diseases [19], [28]. For example, mir-545–3p and mir-34a-5p was utilized as early biomarkers for the diagnosis of pre-clinical AD in the plasma of 40 patients with mild cognitive impairment (MCI) and AD [29].

Currently, the therapeutic approaches for brain disorders or neurodegeneration are limited to the AChE-inhibitors AChEIs; including tacrine (withdrawn), donepezil, rivastigmine, and galantamine, and an N-methyl-D-aspartate (NMDA) receptor antagonist memantine. These anti-AD drugs can improve the symptoms of cognitive impairment; however, they might be associated with side effects including hepatotoxicity [30]. This multi-factorial and complex nature of AD and the variety of involved neuropathogenic pathways hindered the strategy of “one-disease one-target” framework to develop an effective drug, through targeting only one biological target. Therefore, a multi-target directed ligands (MTDLs) approach, with suppression of AChE and Aβ aggregation alongside significant neuroprotection, has attracted interest in developing clinically effective and potential AChEIs [31].

The benzofuran nucleus “2H-1-benzopyran-2-one” constitutes the main structural component of several natural products and synthetic compounds with a plethora of biological activities. Benzofurans are intriguing compounds for the drug discovery in the field of AChEIs; because of the possibility of chemical substitutions at different sites in this core structure.

In our previous publication, the synthetic pathway for the novel benzofuran-based analog was reported [32]. This compound exhibited *in vitro* antioxidant and AChE-inhibitory activities (IC50: 0.058 μM), as compared to the reference drug donepezil (IC50: 0.049 μM). Also, molecular docking results of **compound (IV)** showed good binding modes in the active site of the AChE enzyme, which are similar to the native ligand donepezil [32]. In addition, the biosafety of the compound was studied on different organs at its LD50 concentration and also the behavior response after toxicity induction using AlCl_3_ and treatment with the compound was assessed [32].

Employing an elaborative bio-analytical method for accurate quantification of drugs in different matrix systems is a pre-requisite step in any drug discovery program, and before introduction of a commercial therapeutic agent. Such bio-analytical techniques provide a major role for measuring drug and/or metabolite concentrations in biological fluids, which in turn is required in the assessment of pharmacokinetics (PKs), bioavailability (BA), and bioequivalence (BE) [33], [34], [35]. BA / BE studies have received major attention from academia, the pharmaceutical industry, and health authorities over the last couples of decades.

Hence, the innovation and importance of developing a sensitive and reproducible bioanalytical method tailored for the accurate quantification of new compounds in biological fluids, serves as a cornerstone for regulatory approval and therapeutic success, and also facilitates early decision-making regarding the clinical viability of the studied compound. Such developments not only strengthen the reliability of preclinical and clinical evaluations but also play a decisive role in reducing developmental risks, optimizing therapeutic dosing, and ultimately expediting the successful introduction of new drugs to the market.

The novelty of the present research was to evaluate the potential activity of the new benzofuran-derived” **compound (IV)** in mitigating neuroinflammation, oxidative stress, and synaptic dysfunction; through investigating the expression of a selected panel of specific miRNAs (miR-34a, miR-15a miR-132) in aluminium chloride (AlCl_3_)-intoxicated rats. Moreover, the second part of the study provides the first demonstration of a novel, refined and reproducible UHPLC/UV method for accurate detection and quantification of **Compound (IV)** in the blood circulation (plasma). As a first step towards further research into the corresponding PKs of **compound (IV)** to support drug discovery, the effects of different extraction settings, appropriate column selection, mobile phase composition, flow rate, and detection wavelength were meticulously monitored and optimized.

## 2 Materials and methods

### 2.1 Drugs and chemicals

Aluminum chloride (AlCl_3_) was purchased from Sigma-Aldrich Chemical Company (USA). Donepezil, as a drug of choice against brain disorders was bought from a local pharmacy. ELISA kits were bought from Elabscience (E-EL-R1235) for BDNF and (E-EL-R0015) for interleukin-6 (IL-6). The colorimetric kits for estimating the levels of total antioxidant capacity (TAC), and total protein (TP) content were purchased from Bio-diagnostic Company, Egypt. All chemicals employed for the UHPLC method were of analytical grades, as for methanol (MeOH), and acetonitrile (ACN); they were of HPLC grades acquired from Prolabo, France. Milli-Q deionized water (Millipore Corp., Burlington, MA) was used.

### 2.2 Chemical analysis

This experimental part was performed according to our previous study [32], where melting points, elemental microanalyses, infrared spectra, and 1 H NMR and 13 C NMR spectra and also molecular docking studies were performed at National Research Centre (NRC) and Faculty of Pharmacy-Cairo University, Egypt.

**Scheme:**
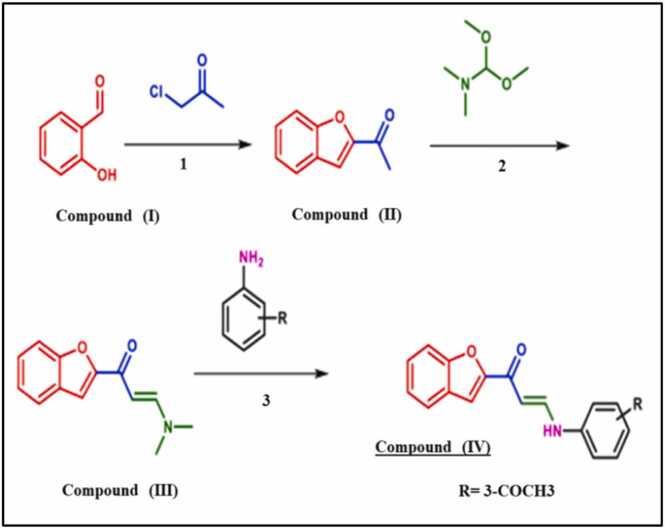
demonstrating the Synthesis of a new benzofuran-based derivative **(Compound IV)** as AChEI for control of Aluminum chloride (AlCl_3_) induced AD-like neurotoxicity; sourced from [32].

#### 2.2.1 *In vivo* study

##### 2.2.1.1 Induction of AlCl_3_ to rats

AlCl_3_ solutions were prepared freshly in distilled water immediately before use, and were administered intragastrically by syringe, to rats daily for two months at a dose of 100 mg/kg/day (0.5 mL per day) [36], [37].

##### 2.2.1.2 Experimental design

Forty adult male Wistar rats weighing 200 ± 20 g were purchased from the animal house of NRC, Egypt. The animals were housed under standard laboratory conditions with a 12 h light/dark cycle (light from 7:00–19:00) and with free access to drinking water and food. This study was conducted according to the principles of the Declaration of Helsinki. The study protocol was approved by the Ethics Committee of NRC (approval number: 19301).

After two weeks of acclimatization, the rats were randomly allocated into four groups (n = 10) as follows:

**Group I:** Normal and healthy rats received no treatments.

**Groups II, III and IV:** AlCl_3_-neurotoxicated rats treated with AlCl_3_ (100 mg/kg/day; orally) for 8 weeks [27], [36].

**Group III:** AlCl_3_-neurotoxicated rats were administered **compound (IV)** (10 mg/kg/day; orally) for 6 weeks [32].

**Group IV:** AlCl_3_-neurotoxicated rats were administered the standard drug “Donepezil” (10 mg/kg/day; orally) for 6 weeks [32].

#### 2.2.2 Blood sampling and preparation

Owing to toxicity induction, the remaining rats after two months of AlCl_3_ intoxication, and six weeks post-treatment amounted to six rats were anaesthetized using thiopental sodium (50 mg/kg, i.p.). Blood was drawn *via* slight rupture of sublingual vein for serum or plasma (using heparinized tubes). Samples were centrifuged using a bench-top centrifuge (Hettich, Germany) at 4000 rpm for 10 min.

#### 2.2.3 Brain tissue sampling and preparation

At the completion of the study, the rats, under anesthesia, were dissected and brains were rapidly excised, cleaned, washed with normal saline and divided longitudinally for molecular and biochemical analyses. Brain samples were homogenized in cold Phosphate Buffer Saline (PBS, pH 7.4). Subsequently, brain homogenates were centrifuged (5000 rpm for 20 min at 4 °C), and the clear supernatants were quickly divided into 100 µl aliquots and stored at −80 °C until use. Brain samples were specified for molecular assays.

#### 2.2.4 Biochemical analyses

##### 2.2.4.1 Estimation of serum IL-6 levels

Serum IL-6 levels were determined by the commercially available ELISA kits (rat) CUSABIO, USA, Catalog No.: CSB-E04640r-1.The optical density of each well was determined within 30 min, using a microplate reader set to 450 nm.

##### 2.2.4.2 Estimation of brain contents of brain-derived neurotrophic factor (BDNF)

The brain contents of BDNF were determined by the commercially available ELISA kits Santa Cruz Biotechnology, Inc. Bergheimer Str. 89–2, 69115 Heidelberg, Germany; catalog no, sc-4554. The optical density of each well was determined, within 30 min, using a microplate reader set to 450 nm.

##### 2.2.4.3 Estimation of serum total antioxidant capacity (TAC)

The evaluation of serum TAC levels is performed by the reaction of antioxidants in the sample with a known amount of exogenous hydrogen peroxide (H_2_O_2_). The antioxidants in the sample eliminate a certain quantity of H_2_O_2_. The residual H_2_O_2_ is estimated by an enzymatic reaction that results in a colored product that could be measured at 505 nm [38].

##### 2.2.4.4 Estimation of brain Total protein (TP) contents

Total protein (TP) was assayed in the serum according to the method of **Bradford** [39].

### 2.3 Molecular study: The microRNA assay: Determination of the differential expression of miRNAs: miRNA-34a, miRNA-15a, and miRNA-132

Prior to RNA extraction, the frozen sera samples were thawed at RT, and total RNA including small RNA was isolated using the miRNeasy kit (Qiagen, Inc., Valencia, CA, USA), according to the manufacturer's instructions. The concentration and purity of the RNA were quantified by measuring the absorbance at 260 nm (A260) and 280 nm (A280) using a NanoDrop spectrophotometer (Thermo Fisher Scientific).

#### 2.3.1 Extraction of miRNAs

MicrRNA was isolated using RNeasy Mini Kit (QIAGEN, Hilden, Germany) according to the manufacturer’s instructions as follow: 1000 μL QIAzol lysis reagent will be added to 200 μL of the plasma sample and then disrupted using vortex. The homogenate will be incubated at room temperature for 5 min 200 μL chloroform is added and the mixture is shaken vigorously. The mixture is centrifuged, and the upper aqueous phase is carefully transferred to a new tube. Cold ethanol is added to this phase, mixed, and the sample is loaded onto an RNeasy Mini spin column. 700 μL RWT buffer will be added to the column then centrifuged for 2 min at ≥ 8000 ×g. and then 500 μL RPE buffer was added to the column and centrifuged for 2 min at ≥ 8000 ×g (this step was repeated twice). Finally, the RNA is eluted with RNase-free water and its concentration is assessed using a NanoDrop Spectrophotometer. For Normalization: Exogenous Controls: Spike-in synthetic miRNAs to monitor extraction efficiency and sample input.

**Reverse transcription (RT) and pre-amplification:** The miRNAs were reverse-transcribed using the TaqMan® microRNA-RT kit (Applied Biosystems, USA) and the associated miRNA-specific stem-loop primers (TaqMan® microRNA assay kit). Total RNA was diluted at a concentration of 12.5 ng/μl and 5 μl of RNA were added to the reaction mix containing 0.15 μl 100 mMdNTP, 1 μl enzyme (50 U/μl), 1.5 μl 10 × RT buffer, 0.19 μlRNase inhibitor (20 U/μl), 1.5 μl 5 × RT specific-primer and 5.66 μl nuclease-free water to obtain a final volume of 15 μl. RT reaction conditions were as follows: 30 min at 16 °C to anneal primers, 30 min at 42 °C for the extension of primers on miRNA and the synthesis of the first cDNA strand, and 5 min at 85 °C to stop the reaction, cDNA was then stored at −80 °C until use.

**Quantitative Polymerase Chain Reaction (PCR) Amplification:** The qPCR reaction mix were prepared (run each sample in duplicates): the reaction mix containing 10 µL 2x TaqMan Universal PCR master mix, 1 µL 20x TaqMan microRNA assay and 8 µL Nuclease free water to obtain a final volume of 19 µL. After mixing, 19 µL of qPCR reaction mix were added to 1.3 µL of RT-reaction (cDNA) in a 96-well plate. Then the plate was transferred to ABI Prism 7900HT. The SDS software was used to set up the run, and then the plates were incubated at 50 °C for 2 min and 95 °C for 10 min, and then 40 cycles of 95 °C for 15 sec and 60 °C for 60 sec were applied.

**Data analysis of the microRNA assay:** The results of the RT-PCR were expressed in terms of cycle threshold (Ct), which is the number of cycles needed for the fluorescent signal to cross the automated threshold. First, ∆Ct was estimated by subtracting the Ct values of the reference RNU6B from the Ct values of the target miRNA for each sample:

“ΔCt = Ct (target) −Ct (reference)”

“ΔΔC t = ΔC t (target) −C t (mean control)”

This is then followed by calculating the fold change, which is equal to 2-ΔΔCT (2 to the power of minus Delta Delta CT).

Normalized target gene expression level (RQ) = 2(−ΔΔC t).

“Δ = delta; CT = threshold cycle; RQ = relative quantification”

#### 2.3.2 Bio-analytical study

##### 2.3.2.1 UV scanning and construction of calibration curve of compound (IV) in methanol

A fresh standard solution of **compound (IV)** in methanol was prepared and scanned spectrophotometrically in the range of 200–800 nm, using UV-Visible spectrophotometer (Beckman, DU-650, USA). Following determination of λmax, the absorbance was plotted against the concentration, and the response factor was calculated. Each concentration was analyzed in triplicate, and the mean values were calculated.

##### 2.3.2.2 UHPLC/UV analysis for detection of compound (IV) in different matrix systems

**Apparatus:** Chromatographic separation was performed on Waters 600 E multi solvent delivery system controller equipped with Rheodyne injector P/N 7725i, and Waters 2487 dual λ absorbance detector coupled to Millennium 32 computer program. Chromatographic separation was achieved using [Symmetry C18, 5 μm, 3.9 cm× 150 mm i.d., Waters]. The column used was protected by a guard pack pre-column module with suitable inserts (*e.g.*, Symmetry C18, 5 μm inserts).

### 2.4 Chromatographic conditions

An isocratic elution scheme was initiated for the chromatographic separation of **compound (IV)**. The mobile phase composition was optimized to get the best possible separation. The elution flow rate varied from 1 to 1.2 mL/min. Column temperature was set at RT, with run time up to 20 min. The effluent was monitored using ultraviolet detection set at different λmax.

### 2.5 Standard solutions

A stock solution of **compound (IV)** (100 μg/mL) was prepared in methanol. A series of standard solutions at different concentrations was serially diluted by the mobile phase to obtain different working solutions.

### 2.6 Extraction procedures

To 0.5 mL rat plasma, quantified amounts of **compound (IV)** were spiked, and vortexed for 30 sec. Two different extraction methods were tried, namely:

(1) Ethyl acetate extraction: 0.5 mL ethyl acetate was added; the mixture was vortexed for 2 min and centrifuged under room temperature (25 °C) for 10 min at 9000 rpm. The upper organic layer was transferred quantitatively and evaporated to dryness in a vacuum concentrator (miVac DUO concentrator, DUC-23050-B00, USA) at 45 °C.

(2) Protein precipitation: 0.5 mL ACN or MEOH was added, followed by vortex for 2 min. Centrifugation step was done either under room temperature (25 °C) for 10 min at 9000 rpm or using cooling centrifugation for 2 min (4000 rpm at 4 °C) (cooling centrifuge, Sigma 3–16KL, Germany). Separation of the supernatant was done quantitatively, followed by evaporation using the vacuum concentrator. The residue obtained from both extraction steps, was reconstituted with 150 μL of the mobile phase, vortexed for 2 min, and then, 50 μL aliquot was injected onto the UHPLC column for analysis.

### 2.7 Method validation

The developed method was validated as per international guidelines in terms of linearity, lower limit of quantification (LLOQ), higher limit of quantification HLOQ, accuracy, precision, and stability parameters [26].

### 2.8 Statistical analysis

Comparisons between the samples were conducted by χ2 or Student's unpaired *t*-test and presented as the mean ± standard deviation (SD). The area under the curve (AUC) using receiver operating characteristic (ROC) analysis was calculated for each miRNA to assess the predictive values. A two-tailed P < 0.05 was considered to indicate statistically significant difference. Also, for biochemical results, statistical analysis was carried out using SPSS (version 19) combined with co-state computer program; biochemical data were presented as Mean ± standard deviation (SD), where different letters are significant at p ≤ 0.05. The percentage of the change, with respect to either the negative control or positive (AlCl_3_-induced) control was calculated according the following formulation: “% Change= (Treated value − Control value) / Control value × 100

## 3 Results

Effect of treatment with compound (IV) on serum levels of IL-6 and TAC, and the brain contents of BDNF and TP in AlCl_3_-induced rats

Herein, our results indicated AlCl_3_ intoxication caused a significant increase in pro-inflammatory serum IL-6 levels and stimulated significant reductions in brain BDNF contents, brain TP contents, and serum TAC levels in AlCl_3_-induced rats, as compared to controls. Treatment of AlCl_3_-induced rats with the **compound (IV)** or Donepezil significantly reduced IL-6 levels along with a significant increment in BDNF levels, and a significant elevation of TAC contents, as compared to induced rats **(**Table 1**)**.

**Table 1:** Effects of treatment of AlCl_3_-induced rats, with either **compound (IV)** or Donepezil, on the serum IL-6 and TAC levels and the brain BDNF and TP contents in different groups.

	Serum IL-6 (*P*g/mL)	Serum TAC (mM/mL)	Brain BDNF (*P*g/g tissue)	Brain TP (mg/dl)
Negative Control	36.50 ±2.31^**a**^	0.90 ±0.03^**a**^	330.10 ± 12.26^**a**^	7.12 ±0.09^**a**^
Neurotoxicated rats	123.25 ±7.00^**b**^	0.20 ±0.01^**d**^	190.59 ±10.90^**b**^	3.03 ±0.07^**d**^
% change to control	**237.67**	**−77.00**	**- 42.26**	**- 57.44**
Neurotoxicated rats + compound IV	58.55 ±5.00 ^**c**^	0.50 ±0.02 ^**c**^	318.76 ±13.54^**a**^	4.90 ±0.06 ^**c**^
% change to neurotoxicated rats	**- 52.94**	**150.00**	**67.25**	**61.72**
Neurotoxicated rats + Donepezil	34.00 ±2.88^**a**^	0.60 ± 0.03^**b**^	343.00 ±16.00^**a**^	5.30 ±0.08^**b**^
% change to neurotoxicated rats	**- 72.41**	**200.00**	**79.97**	**74.92**

Mean ±SD of 6 rats in each group. Statistical analysis was carried out using SPSS computer program, combined with co-state, where different letters are significant at p ≤ 0.05 and similar letters are not significant. IL-6: interleukin-6, BDNF: brain-derived neurotrophic factor, TAC: total anti-oxidant, TP: Total protein. The percentage of the change, with respect to either the negative control or positive (AlCl_3_-induced) control was calculated according the following formulation: “*% Change= (Treated value − Control value) / Control value × 100.*”

### 3.1 Effect of treatment with compound (IV) on the expression of miRNA-34a, miRNA-15a, and miRNA-132 levels in the brain of AlCl_3_-induced rats

**Fig. 1:**
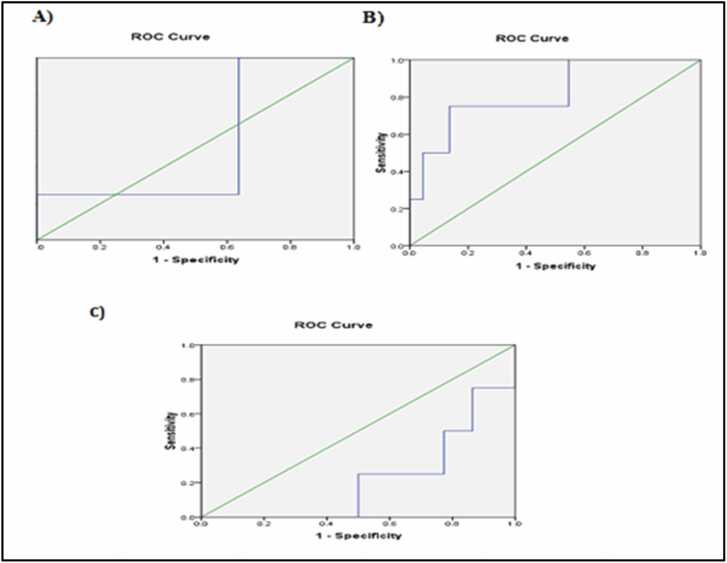
Receiver operating characteristic (ROC) curve analysis for **(A)** miRNA-15a, **(B)** miRNA-132, and **(C)** miRNA-34a. The area under the curve (AUC) using receiver operating characteristic (ROC) analysis was calculated for each miRNA; to assess the predictive values.

AlCl_3_-intoxicated brains exhibited a significant upregulation of miRNA-34a, while levels of miR-15a and miR-132 were significantly downregulated, as compared to the control group (Table 2, Table 3; Fig. 1).

**Table 2:** Effects of treatment of AlCl_3_-neurotoxicated rats, with either **compound (IV)** or Donepezil, on the differential expression of miRNAs (miRNA-34a, miRNA-15a, and miRNA-132) levels in different groups.

	miRNA-34a	miRNA-15a	miRNA-132
Negative Control	0.83 ± 0.02^a^	3.28 ± 0.06^a^	1.41 ±.0.01^a^
Neurotoxicated rats	2.41 ± 0.08 ^c^	0.33 ± 0.01^d^	0.16 ± 0.01^d^
% change to control	**190.36**	**−89.94**	**−88.65**
Neurotoxicated rats + compound IV	1.18 ± 0.06^**b**^	1.86 ± 0.06^**b**^	0.45 ± 0.02^**b**^
% change to neurotoxicated rats	**- 51.04**	**463.64**	**181.25**
Neurotoxicated rats + Donepezil	1.15 ± 0.05^**b**^	2.68 ± 0.05 ^**c**^	0.65 ± 0.04 ^**c**^
% change to neurotoxicated rats	**- 52.28**	**712.12**	**306.25**

Mean ±SD of 6 rats in each group. Statistical analysis was carried out using SPSS computer program, combined with co-state, where different letters are significant at p ≤ 0.05 and similar letters are not significant. The percentage of the change, with respect to either the negative control or positive (AlCl_3_-induced) control was calculated according the following formulation: “*% Change= (Treated value − Control value) / Control value × 100.*”

**Table 3:** Analysis of miRNA-15a, miRNA-132, and miRNA-34a with the corresponding area under the curve (AUC) values.

**Test Result Variable** **(s)**	**AUC**	**Asymptotic Significance**	**Asymptotic 95 % Confidence Interval**	
			**Lower Bound**	**Upper Bound**
**miRNA−15a**	0.523 ± 0.159^**b**^	0.887	0.210	0.835
**miRNA−132**	0.818 ± 0.119 ^**c**^	0.047	0.585	1.052
**miRNA−34a**	0.216 ± 0.111^**a**^	0.051	−0.001	0.433

Mean ±SE of 6 rats in each group. Statistical analysis was carried out using SPSS computer program, combined with co-state, where different letters are significant at p ≤ 0.05 and similar letters are not significant.

### 3.2 Bioanalytical results

#### 3.2.1 UV scanning and construction of calibration curve of compound (IV) in methanol

**Fig. 2:**
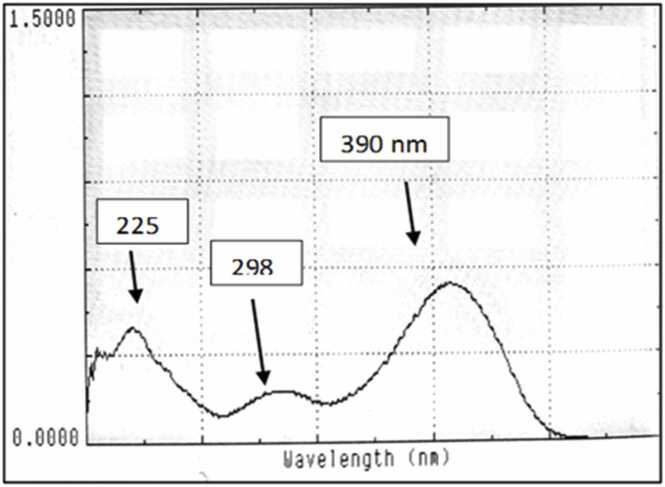
UV-scan of the new anti-AChEI **compound (IV)**.

Fig. 2 showed UV scanning of **compound (IV)** in methanol. The UV-Visible spectrum of **compound (IV)** in methanol was scanned in UV range of 200–800 nm. The wavelengths of maximum absorbance (λmax) were detected at 225, 298 and 390 nm.

**Fig. 3:**
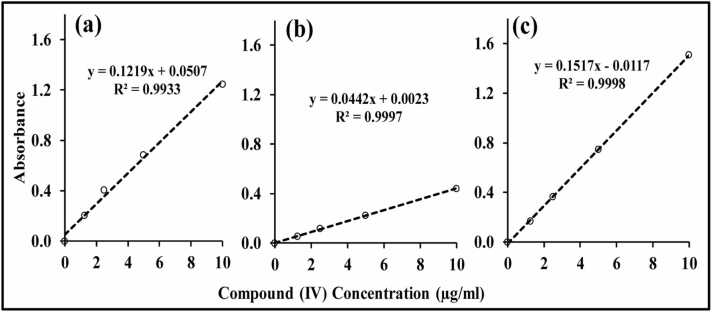
UV Calibration curves of the new **anti-AChEI compound (IV)** in methanol at λ_max_: (a) 225 nm; (b) 298 nm; (c) 390 nm.

Fig. 3 showed the calibration curves of **compound (IV)** in Methanol at 225, 298 and 390 nm. A linear relationship was established with concentration ranges 0.1–10 μg/mL at all predetermined λmax, where the regression coefficient values were 0.9933, 0.9997 and 0.9998, at λmax of 225, 298 and 390 nm, respectively.

### 3.3 Chromatography

Initial trials were conducted to select a suitable entity and type of column, strength of solvent, and optimum flow rate, for the achievement of good separation, better peak shape, and shorter run-time for the studied analyte **“compound (IV)”**.

**a. Column Selection**

**Fig. 4:**
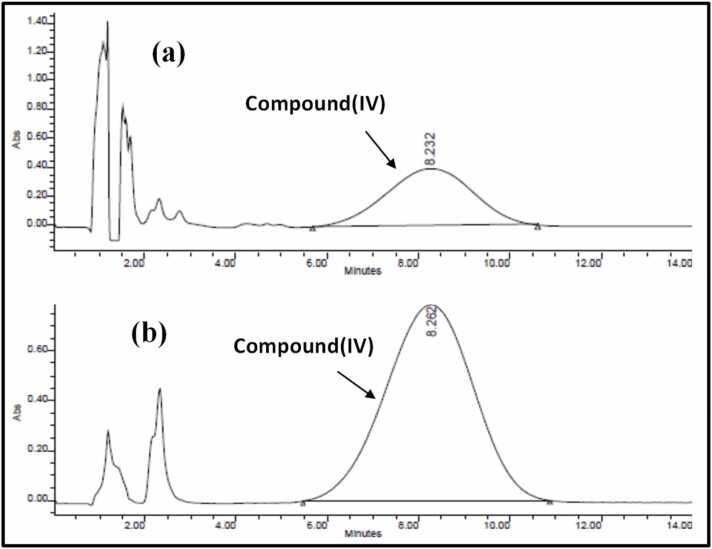
HPLC chromatograms showing peak of **compound (IV)** at 225 nm (a) and 390 nm (b), at retention time of 8.2 min.

Symmetry (C18, 5 μm, 3.9 cm×150 mm i.d., Waters) column was found to give a specific and selective chromatographic separation and was thus selected for the analysis of **compound (IV)**. The mobile phase consisted of ACN: H_2_O (50: 50), adjusted to pH 3 using glacial acetic acid. Chromatographic separation of **compound (IV)** at λmax of 225 nm and 390 nm, with flow-rate of 1 mL/min and retention time of 8.2 min is shown in Fig. 4. Better peak resolution was achieved for λmax of 390 nm, which was selected for further analysis.

**b. Sample Treatment**

**Fig. 5:**
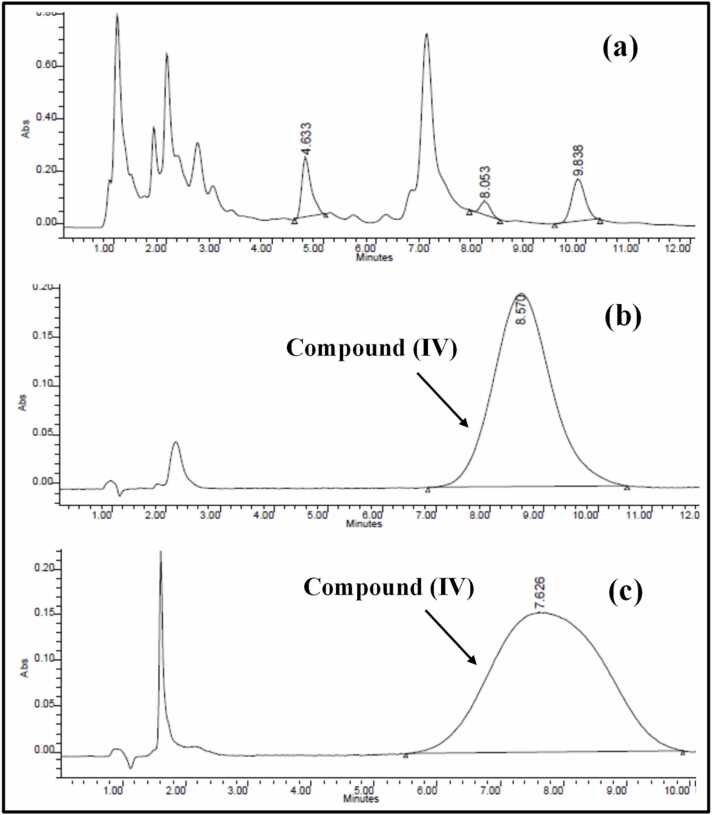
Chromatograms representing spiked **compound (IV)** in rat plasma extracted using: ethyl acetate extraction **(a)**, or protein precipitation method with ACN **(b)**, and MEOH **(c)**.

During the early stage of method development, different extracting solvents were studied, for best separation; results were presented in Fig. 5. It was observed that extraction using ethyl acetate was not successful, with poor extraction efficiency, high noise and many endogenous compounds extracted simultaneously with **compound (IV)**, which gave bad resolution as presented in Fig. 5**a**. On the other hand, Fig. 5 **b & c** represented chromatograms comparing protein precipitation method using ACN or MEOH, respectively. It was clear that ACN provided better resolution with Sharp, narrow peak shape, as depicted in the quality of the resulting chromatograms **(**Fig. 5 **b)**.

**c. Centrifugation Step and Sample Purification**

**Fig. 6:**
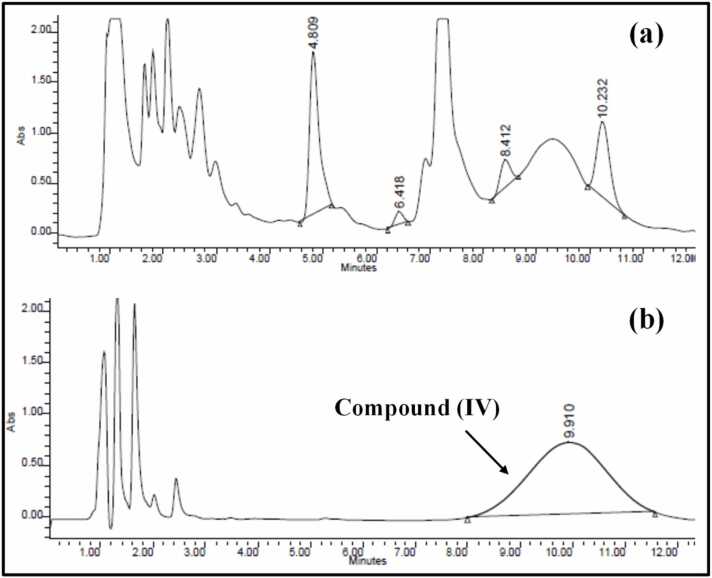
Chromatograms representing spiked **compound (IV)** in the rat plasma adopting the protein precipitation technique using ACN, followed by centrifugation at room temperature (25 °C) **(a) or** cooling centrifugation (4 °C) **(b)**.

Furthermore, samples treated by protein precipitation, using ACN, were centrifuged at RT (25°C)/ 9000 rpm for 10 min or under accelerated centrifugation for 2 min at lower temperature (4°C / 3000 rpm). As presented in Fig. 6**b**, centrifugation at 4 °C resulted in degradation of **compound (IV)** and loss of its characteristic peak.

**d. Mobile phase composition**

### 3.4 Different ratios of organic: aqueous phases

**Fig. 7:**
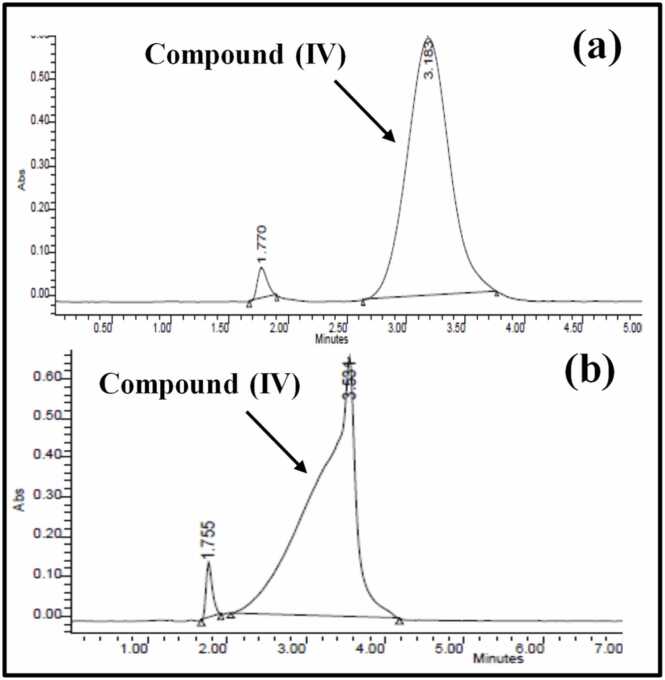
Chromatograms representing spiked **compound (IV)** in the plasma, using ACN: H_2_O at 70:30 **(a)**; 60:40 **(b)**.

Furthermore, two different mixture ratios of ACN: H2O were employed for elution of **compound (IV)** at lower retention time. As presented in Fig. 7, ACN: H2O (adjusted to pH 3 with glacial acidic acid), at ratio (70:30) successfully eluted **compound (IV)** at retention time of 3.2 min, with narrower start- to end- peak better than ACN: H_2_O at ratio (60:40) for the separation of **compound (IV)**.

## 4 Bioanalytical method validation

### 4.1 Linearity, LLOQ & HLOQ

Calibration curves of **compound (IV)** were constructed covering the range of 1–100 μg/mL; the LLOQ & HLOQ for **compound (IV)** were 1 μg/mL and 100 μg/mL, respectively. Detailed results of regression equations with slopes and intercepts for calibration curves were presented in Fig. 8. Linearity of calibration curves was achieved at three levels of 1–5 μg/mL **(**Figs. 8**a)**, 5-25 μg/mL **(**Fig. 8**b)**, and 10–100 μg/mL **(**Fig. 8**c)**, with R^2^ values ≥ 0.99. It could be observed that the developed method provided a wide calibration range (1–100 μg/mL) to enable monitoring expected variations in the plasma **compound (IV)** concentrations.

**Fig. 8:**
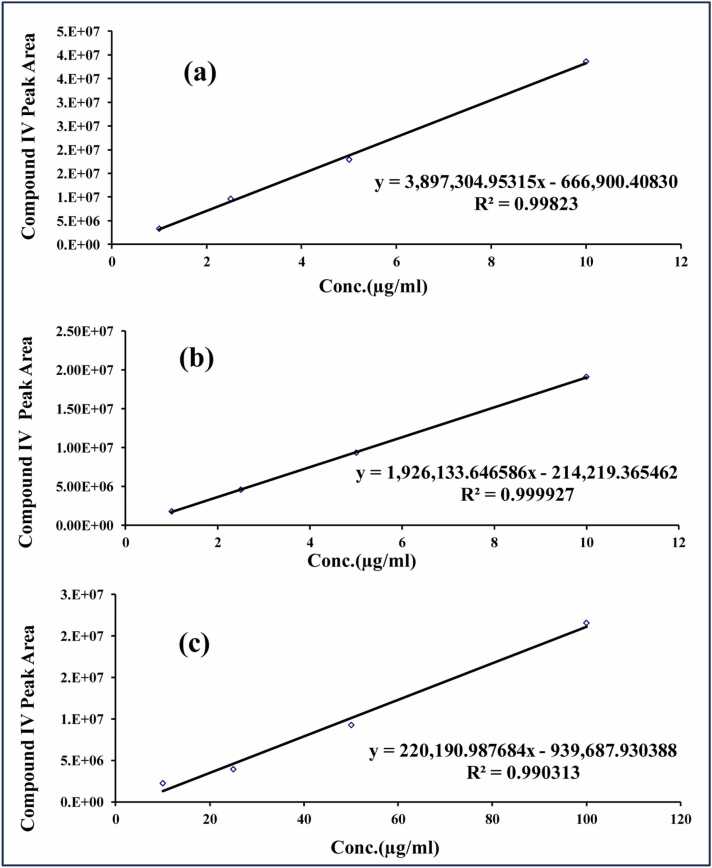
Calibration curves of **compound (IV)** in the rat plasma at concentration range: **(a)** 1–5 μg/mL; **(b)** 5–25 μg/mL; **(c)** 10–100 μg/mL, with detailed regression equations, slopes, and intercepts.

### 4.2 Accuracy and precision

Both parameters were considered intraday (within one day) and interday (between days), for **Compound (IV)** at different concentrations (n = 6). The coefficient of variation (CV %) was used for estimation of precision, while accuracy was evaluated using relative error (RE%). Results revealed that inter- and intraday CV % values were less than 15 %, with inter- and intraday RE% values ranging from 90.12 % to 105.65 %. All precision and accuracy values were in compliance with the acceptance criteria stated in international guidelines [26].

### 4.3 Stability

Results revealed that stock solution of **Compound (IV)** in MEOH was stable for 1 month storage at 4°C. Also, **compound (IV)** plasma samples were stable at room temperature for 24 h. All the aspects of stability studies were satisfactory and proved the validity and robust of the developed method [26].

The analytical features of the promising compound are an initial step for future investigation of its corresponding PKs aiding in drug discovery research. In this study, a precise, simple, inexpensive bio-analytical UHPLC/UV method was successfully developed, with all the chromatographic conditions properly adjusted for sensitive quantification of **compound (IV)** in rat plasma. The new compound was positively eluted, with no interfering or endogenous compounds in rat plasma. This was achieved by different manipulation steps, including proper column selection, constructive extraction steps as well as selective mobile phase composition. The developed method possesses specificity and linearity over the entire range of plasma concentrations, with a LLOQ of 1ug/mL and HLOQ of 100 μg/mL. The developed method required no need for buffers or temperature controller, provided excellent limits of detection, and simplicity of sample preparation involving single step protein precipitation, where, the usual drying followed by reconstitution steps are not needed.

## 5 Discussion

Our results on the potent AChE-inhibitory effect of **compound (IV)** compared with Donepezil was previously demonstrated; the compound showed a safe toxicological profile under the applied dose as evidenced from the previous biochemical and histopathological data on the toxicity of **compound (IV)** on different organs [32]. Therefore, this study aims to investigate its bioanalytical properties, which is the initial step for further *in vivo* PK studies. Furthermore, the current study provides insights into the significant regulatory roles of specific miRNAs in AlCl_3_-induced AD-like pathological mechanisms including neuroinflammation, oxidative stress, and synaptic dysfunction; and presents novel therapeutic and neuromodulatory molecular targets or diagnostic tools related to AD-like pathology.

Herein, our results indicated AlCl_3_ intoxication caused a significant increase in pro-inflammatory serum IL-6 levels (237.67 %) in AlCl_3_-intoxicated rats, as compared to controls. Our results run in agreement with previous studies of **Borai et al.** [3]. This might be attributed to the ability of this neurotoxin (AlCl_3_) to enhance neuroinflammation. Furthermore, in agreement with the present findings, **Khemka et al.** [40] and **Rizk and Aly** [41] found that the levels of pro-inflammatory cytokines, like IL-1β, IL-6 and TNF-α, were elevated in the peripheral circulation in induced neurodegenerative subjects, and are correlated with associated depression and cognitive decline.

Furthermore, AlCl_3_ intoxication induced a significant reduction in brain BDNF contents. Our findings run in concomitant with previous studies [3], [42], [43] that demonstrated significant decreased BDNF levels in induced neurodegenerative subjects. BDNF is involved in the regulation of synaptic plasticity and plays a critical role in maintaining normal cortical function [3]. BDNF exhibits a key role in neurogenesis and synaptic plasticity maintenance [44]. Thereby; reduced BDNF levels is closely associated with the pathophysiology of neurodegeneration through enhancing synaptic dysfunction.

Oxidative stress is strongly correlated with neuroinflammatio; herein AlCl_3_-induced rats exhibited a significant decline in serum TAC levels (77.7 %, as compared to control rats), along with a significant decrease in brain total protein (TP) contents (57.44 %), as compared to control rats; indicating the stimulation of oxidative stress and ROS generation, which enhance downstream pathways related to the up-regulation of apoptotic pathways [37], [45].

Under a prolonged state of chronic neuroinflammation, microglia secretes increased levels of pro-inflammatory cytokines (TNF-α, IL-1β, and IL-6) and stimulates oxidative stress, leading to neurodegeneration [46]. Therefore, the current therapeutic approaches aimed at modulating microglial activation [47]. Treatment of neurotoxicated rats with the **compound (IV)** significantly reduced IL-6 levels (52.94 %) alongside with significant increment in BDNF (67.25 %) contents and significant elevation of TAC (150 %) contents, as compared to induced rats; signifying the potential of **compound (IV)** to exert anti-inflammatory, anti-oxidative, and neuromodulatory activities against AlCl_3_-induced neuroinflammation, oxidative stress, and synaptic dysfunction **(**Table 1**)**.

The current study was also directed to study the effect of **compound (IV)** on regulating the expression of specific miRNAs of therapeutic significance against induced neurodegeneration in rats. The link between miRNAs, oxidative stress, and neuroinflammation in AlCl_3_-induced neurotoxicity indicates the key role for miRNAs in biological responses to induced neurodegeneration. Furthermore, miRNAs are capable of modulating synaptic function and neurotransmission, as demonstrated by their potential involvement in AChE inhibition activity of **compound (IV)**. Certain miRNAs, such as miR-132 and miR-212, are implicated in preserving synaptic integrity and neural signaling through enhancing neurotransmitter levels [46]. Accordingly, the disruption of the expression of these miRNAs is implicated in the reduction of neurotransmitter availability, affecting neural signaling, and finally leading to cognitive decline [20].

Table (2) demonstrated a significant upregulation of miRNA-34a in the neurotoxicated rats (190.36 %), as compared to negative control brains. This runs in agreement with Alexandrov et al. [48], Sarkar et al. [49], and Jian et al. [50] who found that Aluminum exposure upregulates miRNA-34a expression. Over-expression of miR-34a leads to the upregulation of its target genes involved in synaptic plasticity, and oxidative phosphorylation [49]. P53 is a crucial response element of miR-34a; p53/miR-34a axis stimulates apoptosis *via* enhancing caspase-3 and inhibiting Sirt1 and Bcl_2_ expressions in the brains of transgenic APP/PS1 mice [50] another p53-group member Tap73 (p73) stimulates miR-34a expression; through binding certain locations of “miR-34a promoter”. Thus, miR-34a could be regarded as an essential modulator in neuropathogenesis [49].

While miR-15a and miR-132 RQ values, in AlCl_3_-induced brains, were significantly downregulated (89.94, 88.65 %, respectively), as compared to the control group; miR-132 is consistently reduced in AD [51]. MiR-132/212 family members play key roles in neural function and synaptic plasticity, and are continuously down-regulated in early phases of neurodegeneration [52]. Combining detection of miR-206 and miR-132 achieved highest areas under curves (AUC), which is “an index of miRNA’s diagnostic performance” [53]. In addition, these miRNAs might be involved in the impairment of neurogenesis and reduced learning memory ability [54].

This study revealed that the imbalance in the expression of miR-132, miR-15a, and miR-34a, in the neurotoxic brain, is associated with oxidative stress, neuroinflammation, and synaptic dysfunction **(**Table 2**)**. The upregulation of miR-15a [53], [54] and miR-34a is implicated in the accumulation of hyperphosphorylated Tau protein, while downregulation of miR-132 is involved in neuroinflammation [55].

On the other side, treatment of AlCl_3_-induced rats with the **compound (IV)** enhanced a significant downregulation of miRNA-34a (51.04 %), along with significant upregulation of both miRNA-15a and miRNA-132 (463.64 and 181.25 % respectively, as compared to neurotoxicated rats), similarly, treatment with the standard drug (Donepezil) significantly downregulated the expression of miRNA-34a (52.28 %), and significantly upregulated the expression of both miRNA-15a and miRNA-132 (712.12 and 306.25 % respectively, as compared to neurotoxicated rats). The patterns of expression were reversed upon treatment with either **compound (IV)** or the standard drug; this neuroprotective potential was associated with modulation of miR-132, miR-15a, and miR-34a, mitigation of neuroinflammation and oxidative stress, and restoring the synaptic plasticity. It could be concluded that treatment of AlCl_3_-neurotoxicated rats with **compound (IV)** regulated the expression of miR132, miR-15a, and miR-34a in the brain, indicating that regulation of miRNAs expression could be a novel approach to assess the impact of therapeutic agents in the diseased brain.

MiR-132 and miR-212 can inhibit tau phosphorylation by disrupting the balance of S-nitrosylation [56]. It was demonstrated that levels of miR-132 and miR-212 are consistently down-regulated in AD brains, as well as down-regulation of miR-132 in blood [53]. Our research found a significant decrease in miR-132 levels, with about a 9-fold reduction in AD compared to controls; indicating that miR-132, one of the most abundant brain-enriched miRNAs, is critically involved in AD pathogenesis.

Reductions in miR-132 typically occur before neuronal loss, and *in vitro* studies showed that miR-132 protects neurons from deposition of Aβ and glutamate toxicity [57]. Additionally, overexpression of miR-132 has been shown to reduce tau pathology and caspase-3-dependent apoptosis in tau transgenic mice [53]. Decreased miR-132 levels in neural exosomes and blood are associated with cognitive impairment, highlighting its potential as a diagnostic biomarker [16]. The benzofuran-based **Compound (IV)** may exert neuroprotective effects through regulating miR-132, although the specific mechanism of action remains unclear. This compound's potential to modulate miR-132 suggests a promising therapeutic avenue. Further research is needed to elucidate the precise molecular interactions and pathways involved. In addition, miR-15a modulates neuroinflammation, oxidative stress, and autophagy, contributing to neuronal degeneration. Moreover, miR-15a influences synaptic function and neuronal survival by targeting specific mRNAs involved in these processes. Therefore, dysregulation of miR-15a can lead to impaired cognitive function [58].

Collectively, treatment of intoxicated rats with **compound (IV)** improved BDNF and TAC levels, as well as, upregulated the expression levels of both miR-132 and miR-15a; signifying the potential of this synthetic compound to improve the synaptic plasticity and to mitigate oxidative stress and neuroinflammation in malfunctioned brains. This could be ascribed to the ability of miR-132 to bind directly to BDNF and control its expression, this complex regulatory loop constitutes a mechanism by which miRNAs can control their own levels in response to neural activity changes; miR-132 and miR-138, are linked to synaptic formation and function [59]. Also, BDNF participated in controlling the synaptic function. Thus, suppressing miR-132 decreases the increment in BDNF-dependent post-synaptic protein expression. Regarding miR-132, our results run in agreement with several studies [52], [55], that demonstrated that miR-132 is capable of reducing oxidative stress and neuroinflammation *via* p38 signaling pathway. The restoration of miR-132 mitigates the deposition of amyloid and Tau proteins; suggesting its therapeutic potential [60]. In addition, miR-132 upregulation could improve sevoflurane-induced cognitive dysfunction in AlCl_3_-neurotoxicated rats; by suppressing Forkhead-box A1 (FOXA1) [61]. Therefore, identifying certain miRNAs, such as miR-132, implicated in the release of pro-inflammatory cytokines could be a novel therapeutic target [16].

The development of novel AChEIs as targets for controlling brain neuroinflammation is mostly based on the structure-activity relationship (SAR) characteristics of donepezil. Based on the above considerations, to find novel compounds targeting AChE inhibiting activity, the design, synthesis, and biological evaluation of novel derivatives bearing benzofuran nucleus conjugated with a substituted aromatic moiety *via* amino-prop-2-en-1-one linker were previously synthesized [32]. All the new derivatives were examined as AChEIs, utilizing donepezil as a reference drug and **compound (IV)** appeared to possess the most potent and pronounced AChE with IC50 close to that of donepezil (0.58 vs 0.49). The elemental analyses, IR, and NMR of this compound were cited in our previous publication; in addition, molecular docking results of **compound (IV)** showed good binding modes in the active site of the AChE enzyme, which are similar to the native ligand donepezil [32].

The benzofuran-derived **compound (IV)** exerts its effects through molecular interactions with miRNAs, particularly miR-132, miR-34a, and miR-15a, and subsequently influencing their expression and associated pathways. It likely inhibits AChE, increasing ACh levels in the brain, enhancing cognitive function, and reducing AD symptoms. Biochemically, **compound (IV)** was capable of mitigating AlCl_3_-induced neurotoxicity and AD-like pathology through exerting anti-inflammatory activities *via* reducing IL-6 levels, and anti-oxidative activities *via* restoring TAC levels, and demonstrated a restoring potential of synaptic dysfunction *via* increasing BDNF levels. On the molecular levels, downregulation of miR-132 in AD is linked to increased tau phosphorylation and neuroinflammation; hence, restoring miR-132 levels could reduce these pathologies [56]. Similarly, miR-34a regulates apoptosis and neuroinflammation, and its modulation might protect neurons from apoptosis and inflammatory responses [62]. MiR-15a impacts cell cycle regulation and apoptosis, and its modulation could help maintain neuronal health [17]. Overall, by modulating miRNA expression and inhibiting AChE, this compound may offer neuroprotective and anti-inflammatory and anti-oxidant activities, leading to substantial enhancements in synaptic functions. These findings necessitate further mechanistic studies to validate the underlying pathways and the compound's therapeutic potential.

The exact regulatory mechanisms and interactions of these miRNAs in neurodegeneration are not fully understood. In conclusion, while significant progress has been made in understanding the roles of miR-132, miR-15a, and miR-34a, there is still a need for more mechanistic studies to clarify the regulatory mechanisms influencing these miRNA expressions. These studies will not only advance our current knowledge but also pave the way for the development of novel therapeutic strategies targeting these miRNAs.

Based on this, it was of great interest to study the bio-analytical properties of this compound and to investigate its ameliorative impact on certain aspects addressing neurological disorders. The acute and chronic toxicity studies of **compound (IV)** revealed its biosafety since no toxic signs or mortality occurred using different doses of the compound (10, 50, 100 mg/kg b.wt) and thus the standard dose for donepezil was selected in the present study (10 mg/kg b.wt) to allow precise comparison of the compound with the approved drug using the same dosing conditions [32].

This study introduces a novel benzofuran-based compound, 3-((3-Acetylphenyl)amino)-1-(benzofuran-2-yl)prop-2-en-1-one **(IV)**, and investigates its anti-AChE potential. The significance of this research lies in its potential to modulate neuroinflammation and miRNA dysregulation, which are critical factors in neurodegenerative diseases. By administering aluminum chloride (AlCl_3_) as a neurotoxic agent to rats, the current study has discussed key elements in the process of AlCl_3_-induced neurotoxicity; such as neuroinflammation, and ROS generation, which are intertwined with the dysregulation of specific miRNAs, the key molecules that are implicated in the regulatory networks, rendering them as valuable, simple, and less-invasive biomarkers for early detection of neurodegeneration. In addition, the study evaluated the therapeutic potential of **compound (IV)**. This approach is novel as it combines the study of a new chemical entity with the investigation of its effects on neuroinflammation and miRNA expression, providing a comprehensive understanding of its potential benefits in neurodegenerative conditions. The findings could pave the way for developing new treatments targeting the underlying mechanisms of neurodegeneration. Currently, using peripheral miRNA levels as diagnostic biomarkers for neurodegeneration is at advanced phase of clinical development, miRNA profiles in cerebrospinal fluid (CSF), blood, plasma, and serum have been estimated and compared between AD-patients and healthy controls [63], [64].

The present study delivers the first demonstration of a sensitive, reliable and reproducible UHPLC/UV assay for the analysis of **Compound (IV)** in rat plasma. A simple, effective sample treatment method using protein precipitation, together with full optimization of chromatographic parameters: including column selection, mobile phase composition, flow rate and detection wavelength, ensures analyte recovery, accurate quantification, and improve assay sensitivity and reproducibility. The present method provides excellent specificity and linearity over the concentration range of 1–100 μg/mL, as well as low plasma processing volume (50 μL). Furthermore, the proposed method could pave the way for future estimation of primary and secondary PK parameters of **Compound (IV)** in rat plasma, via inclusion of a larger number of rats, as well as study the absorption, distribution, metabolism and elimination (ADME) phases of **Compound (IV)** [33], [34], [35].

This work's practical value goes beyond analytical validation because it guarantees a strong UHPLC/UV platform that directly aids **compound (IV)** translational development. The technique offers the fundamental basis for PK, BA, and dose-exposure investigations, which are crucial for moving a novel entity through the preclinical and clinical development pipeline, by permitting precise and repeatable measurement in plasma. Crucially, the test was created in accordance with worldwide bioanalytical technique validation requirements, such as those set forth by regulatory guidelines [26], [65], guaranteeing both regulatory compliance and laboratory reproducibility. In the end, this proven technique speeds up **compound (IV)**'s possible advancement towards regulatory approval as a therapeutic candidate in addition to facilitating systematic characterization of the compound.

## 6 Conclusion

The current study suggests that the neuroprotective activities of **compound (IV)** such as anti-inflammatory, anti-oxidative activities and its potential to restore synaptic function in AlCl_3_-neurotoxicated brains could be partially ascribed to the restoration of miR-132, expression in the brain and the increment in brain BDNF levels, as well as, the downregulation of both miR-34a and miR-a5a expression in the brain, and the decrement of the pro-inflammatory IL-6. **Compound (IV)** mitigated neurodegeneration; through regulating the expression of miRNAs implicated in the neuropathogenesis of AlCl_3_-induced neurodegeneration such as neuroinflammation, oxidative stress, synaptic dysfunction, and deposition of neurotoxic proteins. Moreover, miR-132a could be regarded as a key player in synaptic plasticity by regulating BDNF levels. In conclusion, the **compound (IV)** complies with Lipinski’s rule of drug-likeness parameters and shows good PK properties. The present study provides a first demonstration of a reliable, linear and reproducible UHPLC/UV method for the quantification of **compound (IV)** in rat plasma, which provides a preliminary step for future generation of adequate PKs of the promising compound. The developed UHPLC method offers good analyte recovery, precise quantification, and improved assay sensitivity and reproducibility, through a straightforward, efficient sample treatment technique that uses protein precipitation in conjunction with optimized chromatographic parameters, including column selection, mobile phase composition, flow rate, and detection wavelength. In addition to having a minimal plasma processing volume (50uL), the current approach offers excellent linearity and specificity over the concentration range of 1–100 μg/mL.

Future challenges will involve expanding validation under different physiological and pathological conditions, and adapting the method for application in long-term PK, BA, and tissue distribution studies. Moreover, comprehensive investigations into potential metabolite detection and eventual clinical applicability will be critical to fully integrate this method into the broader drug development pipeline.

## Limitations

Understanding the mechanisms behind the dysregulation of miRNAs in neurodegenerative diseases is crucial, as these miRNAs and their targets offer significant therapeutic potential for developing treatments. One major challenge in identifying clinically significant miRNAs in diseases is the small sample sizes of clinical studies, which may lack the statistical power to detect meaningful differences in effect size. Additionally, the methods employed to characterize the miRNA profile in each study differ in sensitivity, affecting the outputs. Moreover, the absence of a standardized protocol for miRNA isolation and detection caused identification of non-replicable sets of dysregulated miRNAs. Furthermore, variations in study designs, experimental conditions, study models, sample size, and pharmacological treatments might result in inconsistent outputs. Therefore, more research is required to investigate the PKs of different miRNA *in vivo* to explore the “threshold copies of miRNA”, and to identify the underlying pathological mechanisms of altered miRNA expression levels. Accordingly, further research is required to provide specific cellular or tissue-level evidence, such as quantitative analysis of inflammatory cell infiltration or nerve cell damage.

## Ethical approval

This study was conducted according to the principles of the Declaration of Helsinki. The study protocol was approved by the Ethics Committee of NRC (approval number: 19301).

## Author Contribution Statement

The authors declare that all data were generated in-house and that no paper mill was used. **MZR, KA, LHE, HFA,** and **GIF** conceived and organized the study; **GIF, EY, NE, NFT, ARA MFE** performed experiments and conducted data analysis; **NE, NFT, ARA, MFE, HFA, GIF,** and **MZR** prepared and revised the manuscript.

## Funding

The authors did not receive support from any organization for the submitted work.

## CRediT authorship contribution statement

**Ghadha Ibrahim Fouad:** Writing – review & editing, Writing – original draft, Software, Methodology, Investigation, Formal analysis, Data curation. **Eman Younis:** Investigation, Formal analysis, Data curation. **Maha Zaki Rizk:** Writing – review & editing, Visualization, Validation, Methodology, Investigation, Conceptualization. **Maha Fawzi Emam:** Writing – original draft, Methodology, Investigation, Formal analysis, Data curation. **Khalda Amr:** Resources, Methodology, Formal analysis, Data curation, Conceptualization. **Nesma Elaraby:** Methodology, Investigation, Formal analysis, Data curation. **Laila Hasanin Emara:** Writing – original draft, Methodology, Investigation, Conceptualization. **Hanan Farouk Aly:** Writing – review & editing, Writing – original draft, Visualization, Validation, Methodology, Investigation, Data curation, Conceptualization. **Aya Rashad Abdou:** Writing – original draft, Methodology, Investigation, Formal analysis. **Nesrin Fouad Taha:** Writing – original draft, Methodology, Investigation, Formal analysis.

## Declaration of Competing Interest

The authors declare that they have no known competing financial interests or personal relationships that could have appeared to influence the work reported in this paper.

## Data Availability

Data will be made available on request.
